# Control of acute myeloid leukemia by a trifunctional NKp46-CD16a-NK cell engager targeting CD123

**DOI:** 10.1038/s41587-022-01626-2

**Published:** 2023-01-12

**Authors:** Laurent Gauthier, Angela Virone-Oddos, Jochen Beninga, Benjamin Rossi, Céline Nicolazzi, Céline Amara, Audrey Blanchard-Alvarez, Nicolas Gourdin, Jacqueline Courta, Alexandra Basset, Magali Agnel, Franceline Guillot, Gwendoline Grondin, Hélène Bonnevaux, Anne-Laure Bauchet, Ariane Morel, Yannis Morel, Marielle Chiron, Eric Vivier

**Affiliations:** 1 Innate Pharma, Marseille, France; 2 Sanofi Immuno-Oncology Research, Vitry sur-Seine, France; 3 Sanofi Large Molecules Research, Frankfurt, Germany; 4 Sanofi Drug Metabolism and Pharmacokinetics, Chilly Mazarin, France; 5 Sanofi TMED Biomarkers and Clinical Bioanalysis, Chilly Mazarin, France; 6 Sanofi Preclinical Safety, Chilly Mazarin, France; 7 Sanofi Global Project Management, Vitry sur-Seine, France; 8 Aix-Marseille University, Centre of National Scientific Research (CNRS), National Insititute of Health and Medical Research (INSERM), Centre of Immunology at Marseille-Luminy (CIML), Marseille, France; 9 APHM, Marseille-Immunopole, University Hospital of Timone, Marseille, France

**Keywords:** Cancer immunotherapy, Applied immunology, Immunotherapy, Antibody therapy

## Abstract

CD123, the alpha chain of the IL-3 receptor, is an attractive target for acute myeloid leukemia (AML) treatment. However, cytotoxic antibodies or T cell engagers targeting CD123 had insufficient efficacy or safety in clinical trials. We show that expression of CD64, the high-affinity receptor for human IgG, on AML blasts confers resistance to anti-CD123 antibody-dependent cell cytotoxicity (ADCC) in vitro. We engineer a trifunctional natural killer cell engager (NKCE) that targets CD123 on AML blasts and NKp46 and CD16a on NK cells (CD123-NKCE). CD123-NKCE has potent antitumor activity against primary AML blasts regardless of CD64 expression and induces NK cell activation and cytokine secretion only in the presence of AML cells. Its antitumor activity in a mouse CD123^+^ tumor model exceeds that of the benchmark ADCC-enhanced antibody. In nonhuman primates, it had prolonged pharmacodynamic effects, depleting CD123^+^ cells for more than 10 days with no signs of toxicity and very low inflammatory cytokine induction over a large dose range. These results support clinical development of CD123-NKCE.

## Abstract (web summary)

Leukemia cells resistant to CD123-targeted therapies are susceptible to a trifunctional NK cell engager.

## Main

Acute myeloid leukemia (AML), the most common acute leukemia in adults^1^, is characterized by the clonal expansion of myeloid precursors in the bone marrow (BM) and peripheral blood^2^. There is a clear unmet medical need in AML, as up to 50% of patients relapse after initial chemotherapy^3^, and the prognosis for older patients remains poor^1^.

Several targeted immunotherapies, such as monoclonal antibodies^4^, bispecific T (TCE) and killer cell engager molecules^5,6^ and chimeric antigen receptor (CAR) T cells^7,8^, are currently under clinical evaluation. They target various antigens expressed on AML blasts, with CD33 and CD123 antigens being the most frequently targeted.

CD123, the alpha chain of the interleukin-3 receptor (IL-3Rα), is frequently expressed at high levels in AML^9,10^, mostly on leukemic stem or progenitor cells^11^, associated with a poor prognosis. Cytotoxic antibodies targeting CD123 displayed limited antileukemic activity in several clinical trials^12^, even when specifically engineered to increase antibody-dependent cell cytotoxicity (ADCC)^13^. By contrast, TCE molecules and CAR-T cells have some clinical efficacy^5,14^, but are also highly toxic, confirming the need for alternative targeted approaches.

NK cell-based therapies may provide new treatment perspectives and a safer alternative for targeting AML cells in this context^15–18^, without the complications frequently associated with T cell therapies, such as cytokine release syndrome or neurotoxicity^19^. NK cells are innate lymphoid cells that can recognize and kill virus-infected cells or cancer cells^20–22^. Several activating receptors can be targeted to induce NK cell-mediated antitumor immunity^23^, including CD16a (FcγRIIIa), NKG2D, and the natural cytotoxicity receptors (NCRs) NKp30 and NKp46 (refs. 24–26). Because the full activation of NK cells requires the coengagement of different activating receptors^27,28^, we have developed an antibody-based NK cell engager (NKCE) technology for the generation of trifunctional molecules (NKp46-CD16a-NKCEs) targeting antigens expressed on cancer cells and coengaging NKp46 and CD16a on NK cells^29,30^. NKp46 (NCR1, CD335) is an activating cell-surface glycoprotein highly conserved in mammals^25^. NKp46 is expressed on all NK cells^31^, ILC1 and very small T cell and ILC3 subsets^32^. NKp46 signaling is mediated by association with CD3ζ and FcRγ, which trigger NK cell activation, cytotoxicity and cytokine release^33^. The development of AML blasts in bone marrow affects normal hematopoiesis and the development of immune cells, including NK cells^34^. NK cells from patients with AML often express low levels of NKp46 at diagnosis^35^, but induction chemotherapies can restore NK cell function and normal NKp46 expression^36^, and high levels of NKp46 at the cell surface correlate with better outcomes in allogeneic stem cell transplantation in patients with AML^37^.

The genetic heterogeneity of AML can also translate into complex expression profiles for various cell-surface markers^38^ including the Fc-gamma receptors (FcγRs) CD16, CD32 and CD64 (ref. 39). CD64 (FcγRI) is a high-affinity receptor for human IgG expressed on healthy monocytes and macrophages^40^; it is expressed on AML blasts in about one-third of patients^38,39^.

We report here the development of a trifunctional NKCE molecule (CD123-NKCE) targeting CD123 on AML cells. We observed that the expression of CD64 on AML cells from patients inhibited the ADCC induced by antibodies targeting CD123 in vitro and ex vivo, but had no effect on CD123-NKCE, which displayed potent antitumor activity against primary malignant AML blasts and cell lines regardless of CD64 expression.

## Results

### CD64 expression on AML cells inhibits anti-CD123 antibody ADCC

We investigated whether NKp46-based NKCE technology could provide more effective antitumor activity than regular IgG antibodies for AML treatment. We generated a NKCE molecule targeting CD123, evaluated the ex vivo antitumor activity of this molecule, and compared it with an antibody derived from clone 7G3 (CD123-IgG1^+^)^41^ engineered for enhanced ADCC^42^.

**Fig. 1:**
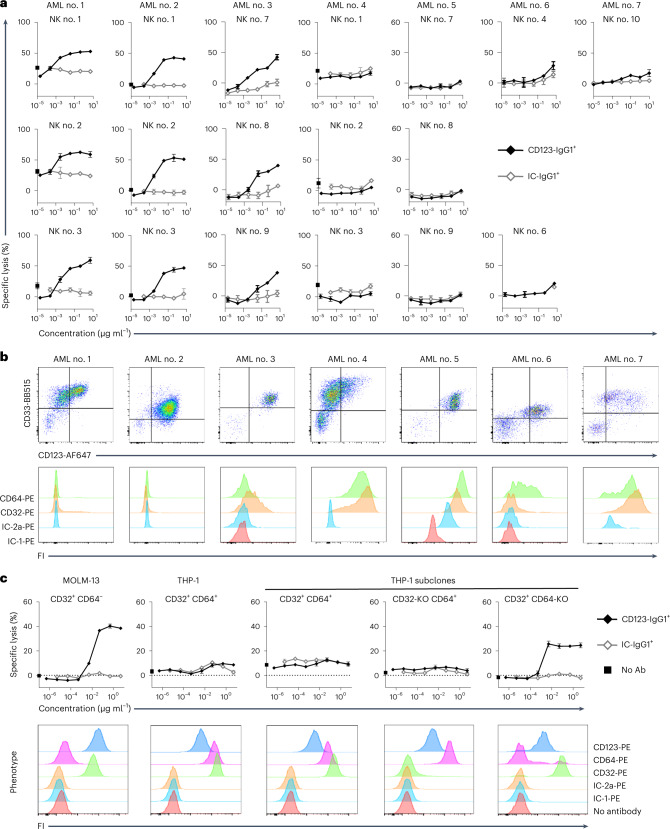
The expression of high-affinity FcγR CD64 on AML cells inhibits the ADCC activity of the anti-CD123 antibody in vitro. **a**, Cytotoxicity of the anti-CD123 antibody (CD123-IgG1^+^) against AML blasts from patients. Malignant cells from seven patients with AML were used as targets and purified NK cells from ten healthy donors were used as effectors. Results are shown for all healthy donor NK cells tested. **b**, Phenotype of the malignant AML cells from patients used in **a** showing the expression of CD33, CD123, CD32a/b and CD64. FI, fluorescence intensity. **c**, Upper panels show the cytotoxicity of anti-CD123 antibody (CD123^−^IgG1^+^) against AML cell lines with and without expression of CD32 and CD64. MOLM-13 (CD32^low^CD64^−^) and THP-1 (CD32^+^CD64^+^) cells and THP-1 subclones with inactivated CD32 (CD32-KO CD64^+^) or CD64 (CD32^+^ CD64-KO) expression were used as the target cells, with purified resting NK cells from healthy donors as effectors (*n* = 3). Data of **a** and **c** are presented as mean values ± s.d. Lower panels show the phenotype of the AML cell lines expressing CD123, CD32 and CD64. Ab, antibody.

The anti-CD123 antibody-mediated killing of primary blasts from patients with AML (AML no. 1 to AML no. 7) was evaluated ex vivo with NK cells from healthy donors as effectors (Fig. 1a). The anti-CD123 antibody (CD123-IgG1^+^) mediated the killing of blasts from about half the samples from patients (AML no. 1 to 3; Fig. 1a) but was barely active against blasts from the other half of samples (AML no. 4 to 7; Fig. 1a), therefore separating samples into two groups: CD123-IgG1^+^-responders and CD123-IgG1^+^-nonresponders. The difference between these two groups cannot be accounted for by simple differences in the expression of CD123, as samples from patients with similar CD123 levels were distributed between the two groups (Fig. 1b). One notable difference between the two groups was the absence of FcγR expression on the AML blasts of CD123-IgG1^+^-responders and its presence in CD123-IgG1^+^-nonresponders, whose cells expressed CD32 (a and/or b isoforms) and/or CD64 (Fig. 1b). We therefore hypothesized that the expression of the FcγRs CD32 and/or CD64 on malignant AML cells might interfere with ADCC, by sequestering the antibody Fc in *cis* at the surface of CD123^+^FcγR^+^ target cells. We tested this hypothesis by evaluating the killing activity of CD123-IgG1^+^ further with two standard AML cell lines, MOLM-13 and THP-1, which express CD123 at similar levels but differ in their expression of FcγRs (Fig. 1c). MOLM-13 cells had much lower levels of CD64 and CD32 than THP-1 cells. CD123-IgG1^+^ was active against MOLM-13 cells (CD32^low^, CD64^-/low^) and completely inactive against THP-1 cells (CD32^+^; CD64^+^) cells. For clarification of the potential respective roles of CD32 and CD64 in resistance to CD123-IgG1^+^ killing, we selectively knocked down the expression of CD32a, CD32b and CD64 in THP-1 cells. We then evaluated CD123-IgG1^+^ killing activity on THP-1 subclones expressing CD32 only, CD64 only, or both CD32 and CD64 (Fig. 1c). We found that CD64 played a dominant role in resistance to ADCC, as CD123-IgG1^+^ killing activity was restored only in the absence of CD64 expression. These results support the hypothesis that the *cis* capture of antibody Fc by high-affinity FcγR CD64 at the surface of the target cell interfered with ADCC, probably by competing with *trans* binding to CD16a on NK cells.

### CD123-NKCE overcome CD64-mediated inhibition of AML killing

Unlike cytotoxic antibodies, NKCE molecules engaging NKp46 can promote NK cell cytotoxicity in a CD16a-independent manner^29^. We therefore explored whether NKCE molecules engaging only NKp46 or coengaging both NKp46 and CD16a, could induce the NK cell-mediated killing of CD64-expressing AML target cells.

**Fig. 2:**
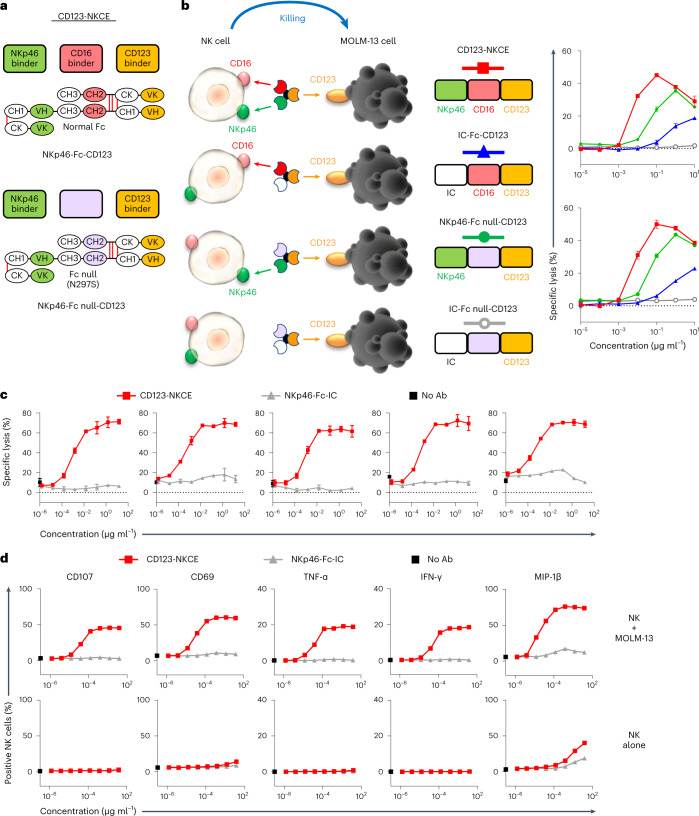
CD123-NKCE displays strong cytotoxic activity against AML cells, strong activation of NK cells and no off-target effects. **a**, Diagrams showing the molecular organization of the NKCE molecules. The top shows the CD123-NKCE trifunctional molecule built with an unmodified human IgG1-Fc (red), targeting CD123 (orange) and coengaging NKp46 (green) and CD16a on NK cells. The bottom shows the bifunctional NKCE containing a human IgG1-Fc silenced for binding to all FcγRs (Fc null; purple). **b**, Comparison of the cytotoxicities of NKCEs targeting CD123 and engaging CD16a only (IC-Fc-CD123), NKp46 only (NKp46-Fc null-CD123) or coengaging NKp46 and CD16a (NKp46-Fc-CD123; CD123-NKCE). MOLM-13 cells were used as the targets and purified resting NK cells as effectors. Results for two healthy donors are shown (*n* = 3). Data are presented as mean values ± s.d. **c**, Cytotoxicity of CD123-NKCE against the AML cell line MOLM-13. Results for five healthy donors are shown. Data are presented as mean values ± s.d. **d**, Evaluation, by flow cytometry, of CD107, CD69, TNF-α, IFN-γ and MIP-1β expression by NK cells treated with CD123-NKCE. NK cells alone are compared with NK cells cocultured with MOLM-13 cells. Results for one representative donor are shown (*n* = 3).

NK cell engager molecules targeting CD123 on AML cells and engaging NKp46 (NKp46-Fc null-CD123), or coengaging NKp46 and CD16a (NKp46-Fc-CD123: CD123-NKCE) on NK cells (Fig. 2a and Supplementary Figs. 1 and 2) were generated. NKp46-Fc null-CD123 was built with a silenced version of human IgG1-Fc (Fc null) mutated at position 297 (EU-numbering). The Fc of NKp46/CD16a coengager molecule (CD123-NKCE) was not modified and binds all FcγRs with regular affinity (Extended Data Table 1). Anti-NKp46 and anti-CD123 antibody moieties bind to human NKp46 and CD123 with monovalent dissociation constant (*K*_D_) of 16.6 ± 1.1 and 0.40 ± 0.02 nM, respectively (Extended Data Table 1).

As already described for other target antigens and cancers^29^, a bifunctional NKp46-NKCE targeting CD123 (NKp46-Fc null-CD123) had strong antitumor effects against the MOLM-13 AML cell line in vitro (Fig. 2b). The coengagement of NKp46 and CD16a with trifunctional NKCEs potentiated NK cell activation (Fig. 2b), CD123-NKCE demonstrating potent killing activity (geometric mean half-maximum effective concentration (EC_50_) of 4.2 (95% confidence interval (CI): 2.9, 6.3) pM, and mean observed maximum specific lysis of 71 ± 5%) and good consistency between NK cells from healthy donors (Fig. 2c). Moreover, we confirmed that CD123-NKCE activated NK cells and promoted the expression of the activation marker CD69, degranulation marker CD107a, TNFα, IFN-γ and MIP-1β effector cytokines/chemokines in a dose-dependent manner, exclusively in the presence of target cells expressing CD123 (Fig. 2d).

**Fig. 3:**
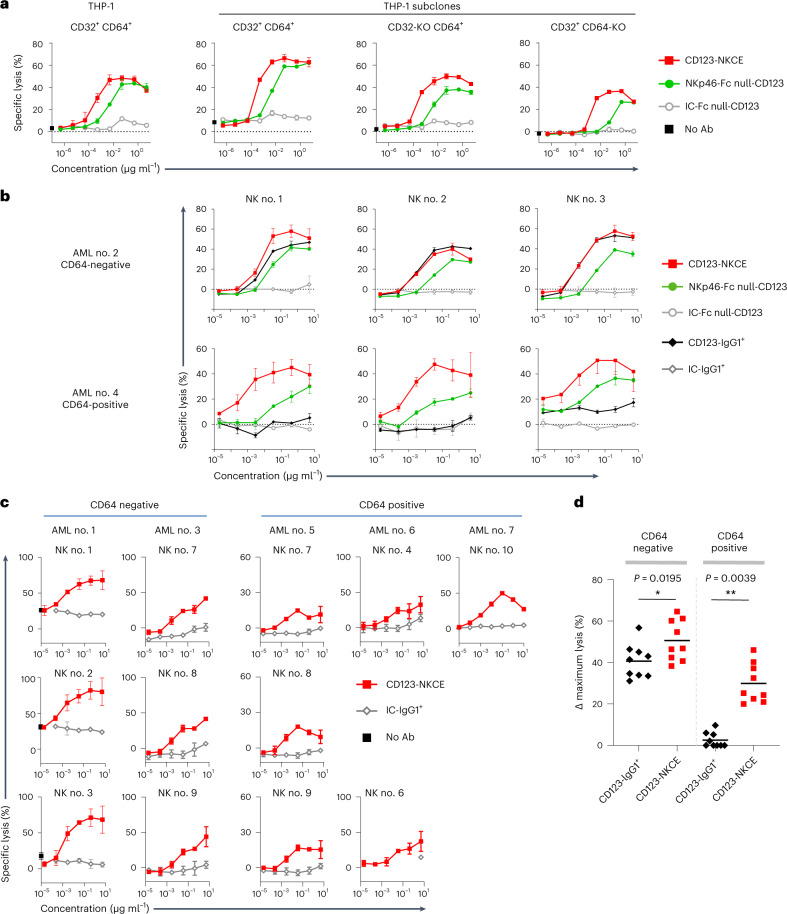
CD123-NKCE displays strong cytotoxic activity against AML cells that is not affected by expression of CD64. **a**, Comparison of the cytotoxicities of NKCE molecules engaging only NKp46 (NKp46-Fc null-CD123) or coengaging NKp46 and CD16 (CD123-NKCE) against AML cell lines with and without CD32 and CD64 expression. THP-1 (CD32^+^ CD64^+^) cells and THP-1 subclones with inactivated CD32 (CD32-KO CD64^+^) or CD64 (CD32^+^ CD64-KO) expression were used as the targets, and purified resting NK cells from healthy donors were used as effectors. The data of one representative experiment among three are shown. **b**, Comparison of the cytotoxicities of CD123-IgG1^+^(black lozenge), NKp46-Fc null-CD123-NKCE (green circle) and CD123-NKCE (red square) against primary AML blasts expressing or not CD64. Primary AML blasts CD64-negative (AML no. 2) and CD64-positive (AML no. 4) were used as the targets and purified resting NK cells as effectors. The data for three healthy donor NK cells are shown. **c**, Cytotoxicity of CD123-NKCE against blasts from patients with AML. five blasts from patients with AML (AML nos. 1, 3, 5, 6 and 7) were used as targets, and purified NK cells from healthy donors (*n* = 9) were used as effectors. Results are shown for all the healthy NK cell donors tested. Data of **a** and **c** are presented as mean values ± s.d. **d**, Maximum cytotoxic activities of CD123-IgG1^+^ and CD123-NKCE molecules against blasts from patients with AML. Blast cells from seven patients with AML were used as targets and purified NK cells from ten healthy donors were used as effectors. Delta (Δ) maximum lysis, defined as percentage maximum lysis of the compound minus percentage background lysis of the isotype control molecule (IC-IgG1^+^ or IC-NKCE), were monitored from the dose response of each compound, and plotted separately for all couples of primary CD64-positive and CD64-negative AML sample/NK donor. (*P ≤ 0.05; **P ≤ 0.005; two-sided Wilcoxon matched-pairs signed rank test).

Unlike anti-CD123 antibody, both bifunctional and trifunctional NKp46 engager molecules mediated strong killing of CD64-positive THP-1 cells (Fig. 3a). We observed that trifunctional CD123-NKCE was equally potent against the parental THP-1 cells, THP-1 subclones and MOLM-13 cells, regardless of CD64 expression status on target cells (Figs. 2c and 3a).

In addition, we also compared the activity of NKp46-Fc null-CD123 and CD123-NKCE on CD64-negative and CD64-positive primary sample AML no. 2 and AML no. 4 (Fig. 3b). We observed that bifunctional and trifunctional NKCE were active on both CD64-negative and CD64-positive AML samples, and that CD123-NKCE was consistently more potent than the bifunctional molecule.

Moreover, trifunctional NKCE molecules also displayed killing activity against all primary AML cells (Fig. 3c), promoting significant antitumor activity in CD64-positive samples from patients with AML (AML nos. 4–7) against which the regular anti-CD123 cytotoxic antibody was completely inactive. The maximum cytotoxic activities (Δ maximum lysis) of CD123-IgG1^+^ and CD123-NKCE were compared on both CD64-positive and CD64-negative primary AML groups (Fig. 3d). We observed that CD123-NKCE molecule was significantly superior to CD123-IgG1^+^ on CD64-negative samples on which the IgG1 was nevertheless active (*P = 0.0195), and highly superior to CD123-IgG1^+^ on CD64-positive samples on which the latter was inactive (**P = 0.0039).

**Extended Data Fig. 1:**
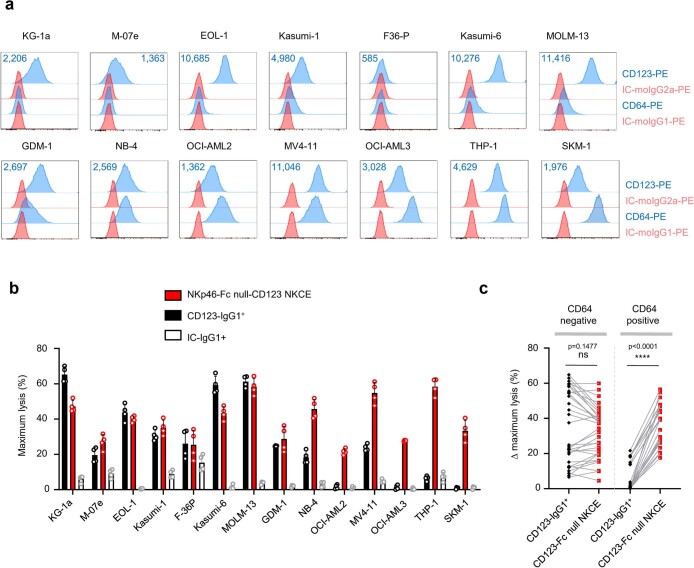
CD123^−^NKCE promotes strong killing activity against AML cells that is not affected by the expression of CD64. **a**, Phenotype of the 14 AML cell lines used in the study showing the expression of CD64 and CD123 at the cell surface by flow cytometry. Antibody binding capacity (ABC) values for CD123 are indicated. **b**, Maximum cytotoxic activities of the anti-CD123 antibody (CD123-IgG1+; black), NKp46-Fc null-CD123 NKCE (red), and IgG1 isotype control (IC-IgG1+; white) against AML cell lines. AML cell lines were used as targets and purified NK cells from 4 healthy donors were used as effectors. Data are presented as mean values ± s.d. **c**, Delta (Δ) maximum lysis, defined as percent maximum lysis of the compound minus percent background lysis of the isotype control molecule (IC-IgG1^+^) at the corresponding concentration, were monitored from the dose response of each compound, and were plotted separately for all couples of CD64^−^positive and CD64-negative AML cell lines/NK donor. (ns P > 0.05; **** P ≤ 0.0001; two-sided Wilcoxon matched-pairs signed rank test).

The superiority of CD123-NKCE was confirmed on a large panels of AML cell lines (Extended Data Fig. 1a–c) coexpressing or not CD64 at the cell surface and expressing CD123 at various cell-surface densities with antibody binding capacity ranging from 580 to more than 10,000 antibody sites per cell (Extended Data Fig. 1a).

For all the CD64-negative or low cell lines (that is, KG-1a, M-07e, EOL-1, Kasumi-1, F36-P, Kasumi-6, MOLM-13 and GDM-1) the Δ maximum killing activity was comparable between CD123-IgG1^+^ and NKp46-Fc null-CD123 molecule (Extended Data Fig. 1b,c). NKCE molecule was consistently active on all CD64-positive AML cell lines (that is NB-4, OCI-AML2, MV4-11, OCI-AML3, THP-1 and SKM-1) whatever the CD64 density of expression, with maximum killing activity globally comparable to those observed for CD64-negative or low cell lines. On the contrary, CD123-IgG1^+^ was completely inactive on OCI-AML2, OCI-AML3, THP-1 and SKM-1 CD64-positive cell lines, and showed limited activity on NB-4 and MV4-11 compared to the NKCE that was significantly highly superior to CD123-IgG1^+^ (Extended Data Fig. 1c, *P* < 0.0001), confirming with AML cell lines the results observed on primary AML samples.

**Extended Data Fig. 2:**
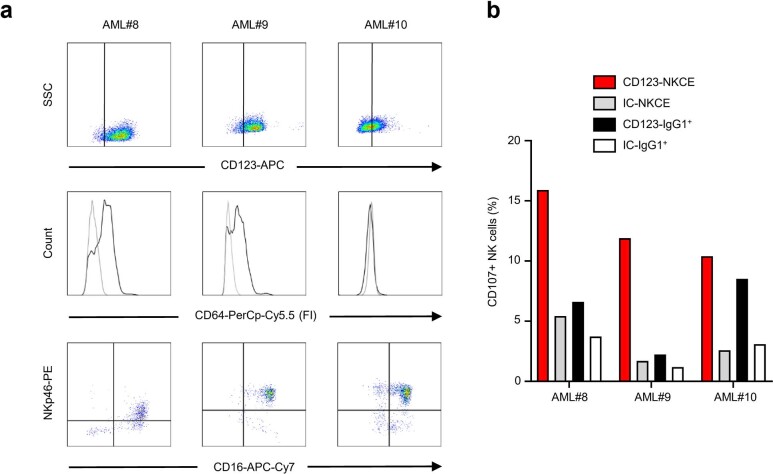
CD123^−^NKCE-mediated activation of autologous NK-cells against AML blasts is not affected by the expression of CD64. **a**, Phenotypes of NK and malignant cells from AML patients. *Upper panels*, Expression of CD123 on AML blasts (gated on the CD33^−^positive population); *middle panels*, expression of CD64 (CD64 staining in black and isotype control in gray) on CD123-positive AML blasts; *lower panels*, expression of NKp46 and CD16a on AML sample NK cells. **b**, Measurement, by flow cytometry, of CD107a/b expression by NK cells after the overnight treatment of PBMCs from AML patients with 120 ng/mL CD123-NKCE (red), anti-CD123 antibody (CD123-IgG1^+^; black), and IC-IgG1^+^ (white) and IC-NKCE (gray) negative control molecules.

An autologous NK cell activation assay performed with additional samples from patients with AML, two CD64-positive (AML nos. 8 and 9) and one CD64-negative (AML no. 10) (Extended Data Fig. 2a,b), further confirmed that, unlike CD123-IgG1^+^, which was active only against the CD64-negative sample (AML no. 10), CD123-NKCE mediated the autologous activation of NK cells from the three patients against their own blasts regardless of CD64 expression (Extended Data Fig. 2b).

### CD123-NKCE controls AML tumor growth in vivo

**Fig. 4:**
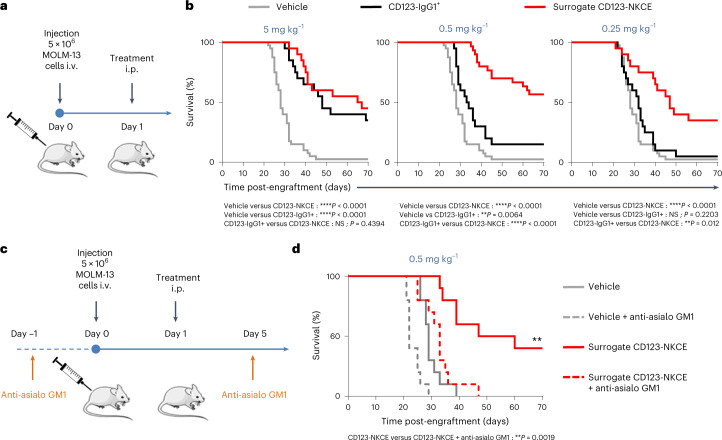
CD123-NKCE promotes tumor growth control in vivo. **a**, Schematic diagram of the experimental setting used in **b**. i.p., intraperitoneal. **b**, Mice engrafted with MOLM-13 cells i.v. were treated, on the day after cell injection, with 5 mg kg^−1^ (left panel), 0.5 mg kg^−1^ (middle panel) or 0.25 mg kg^−1^ (right panel) surrogate CD123-NKCE (red), anti-CD123 antibody (CD123-IgG1^+^; black), or vehicle (gray). Kaplan–Meier curves were plotted for the analysis of mouse survival. Endpoint significance was calculated in a log-rank test. *n* = 10 to 20 per group. *P < 0.05; **P < 0.01; ***P < 0.001;****P < 0.0001. NS, not significant. **c**, Schematic diagram of the experimental setting used in **d**. **d**, Mice were split into two groups, one treated with anti-asilo GM1 1 day before engraftment (day −1) and on day 5 to deplete NK cells, and the other left untreated. MOLM-13 cells were transplanted i.v. into the mice of the two groups on day 0, and the mice were then treated, the day after cell injection, with 0.5 mg kg^−1^ surrogate CD123-NKCE (red) or vehicle (gray). Kaplan–Meier curves were plotted to analyze mouse survival. Dashed lines correspond to the groups treated with anti-asialo GM1 antibody. Endpoint significance was calculated in a log-rank test. *n* = 10 per group. *P < 0.05; **P < 0.01; ***P < 0.001; ****P < 0.0001. NS, not significant.

We then assessed the in vivo efficacy of trifunctional CD123-NKCE in a xenogeneic disseminated AML tumor model induced by the intravenous (i.v.) injection of MOLM-13 tumor cells. We used a surrogate trifunctional molecule targeting the previously validated 29A1.4 epitope of mouse NKp46 (refs. 29, 43) and human CD123 (Fig. 4a,b). The surrogate CD123-NKCE was more effective than the comparator anti-CD123 antibody over a range of doses, with 40 and 60% of mice rescued from death at doses of 0.25 and 0.5 mg antibody per kilogram body weight, versus no mice rescued at these doses in the CD123-IgG1^+^-treated groups. CD123-NKCE was also more effective than CD123-IgG1^+^ at the highest dose (5 mg kg^−1^), with 45% of mice rescued and a 135% increase in lifespan in the NKCE group, versus 35% of mice rescued and an increase in lifespan of 70% in the antibody group (Fig. 4b). The depletion of mouse NK cells by treatment with anti-asialoGM1 antibodies totally abolished CD123-NKCE efficacy in that model (Fig. 4c,d), confirming the major role of NK cells in the antitumor activity of trifunctional NKCE molecules in vivo.

### CD123-NKCE is active and safe ex vivo and in nonhuman primates

**Fig. 5:**
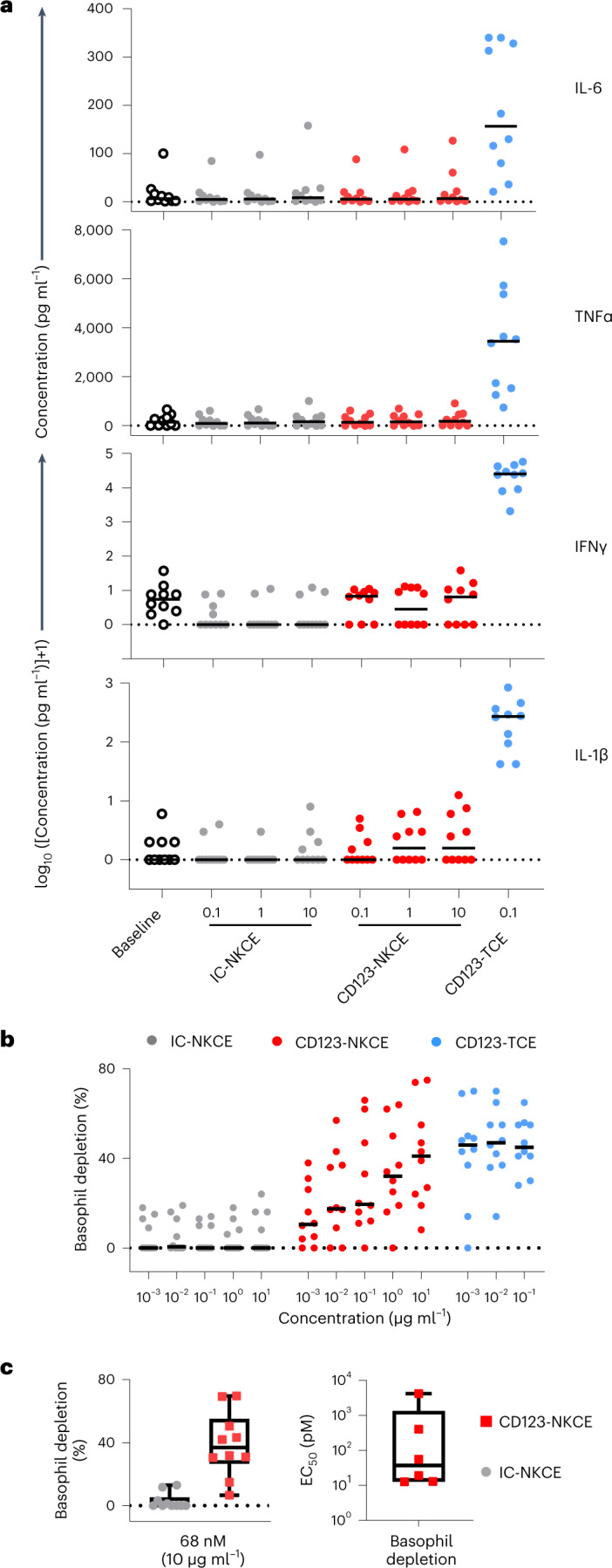
CD123-NKCE mediates pharmacodynamics effects in human PBMCs with negligible cytokine release as compared to CD123-TCE. **a**, IL-1β, TNF-α, IFN-γ and IL-6 cytokine release in vitro by PBMCs from healthy donors (*n* = 10) following the administration of CD123-NKCE (dose range 0.1 to 10 µg ml^−1^), control NKCE, or a bispecific T cell engager tool targeting the same antigen (CD123-TCE, 0.1 µg ml^−1^). Individual (dot) and median (bar) values are shown. **b**, CD123-positive basophil depletion activity in healthy donor PBMCs (*n* = 10) following the administration of CD123-NKCE (dose range 0.001 to 10 µg ml^−1^), control NKCE (IC-NKCE) or CD123-TCE (dose range 0.001 to 0.1 µg ml^−1^). **c**, Left panel shows a boxplot, with whiskers showing minimal and maximal value and upper and lower quartile box limits, of CD123-NKCE maximum depletion activity of the ten donors at the highest dose tested (10 µg ml^−1^, 68 nM). Right panel shows a boxplot, with whiskers showing minimal and maximal value and upper and lower quartile box limits, of EC_50_s for CD123-positive basophil depletion calculated from CD123-NKCE dose responses for six healthy donors among ten.

Potent cytotoxicity may be associated with toxicity in patients. We therefore measured cytokine release from human peripheral blood mononuclear cells (PBMCs) induced by CD123-NKCE in vitro, comparing the results with those of a CD3 T cell engager antibody tool (CD123-TCE) targeting the same antigen^5^. PBMCs of healthy donors (*n* = 10) were cultured for 20 h in the presence of CD123-NKCE or CD123-TCE, and the secretion of IL-6, IL-1β, TNF-α and IFN-γ was quantified (Fig. 5a).

CD123-NKCE induced much lower levels of cytokine release than CD123-TCE, even at concentrations that were 42 times higher (Fig. 5a).

CD123 is expressed on a subset of circulating basophils and plasmacytoid dendritic cells (pDC). Given the low abundance of pDCs among human PBMCs, we focused on basophils to monitor the depletion of CD123^+^ cells by flow cytometry in the same assay (Fig. 5b,c). The treatment of PBMCs with CD123-NKCE promoted a dose-dependent partial depletion of CD123^+^ basophils with a median maximum depletion of 37% (31; 50), and a median EC_50_ value of 38 pM (95% CI (13; 408)), calculated with six of ten donor samples (Fig. 5b,c). Thus, effective NK cell activation and recruitment by CD123-NKCE were associated with a pharmacodynamics effect of CD123^+^ cell depletion in human PBMCs, but without marked proinflammatory cytokine release at up to 10 µg ml^−1^ dose (68 nM), suggesting that NKCEs have a better benefit/risk profile than TCEs for the treatment of AML.

We further performed dedicated pharmacokinetic, pharmacodynamics and toxicology studies in nonhuman primates (NHPs). Cynomolgus monkeys were selected as a relevant species for these studies on the basis of their tissue distributions of NKp46 and CD123, which are similar to those in humans^43,44^, and because the CD123-NKCE molecule binds to cynomolgus antigens and Fc receptors with affinities similar to those of human (Extended Data Table 1). In addition, a regulatory cross-reactivity study^45^ performed by immunohistochemistry with the CD123-NKCE molecule on panels of human and cynomolgus normal tissues, confirmed similar staining distribution in endothelial cells of vessels and in mononuclear cells in many organs on both species, with tissue localization similar to those previously reported for NKp46 and CD123 antigens^46,47^.

**Fig. 6:**
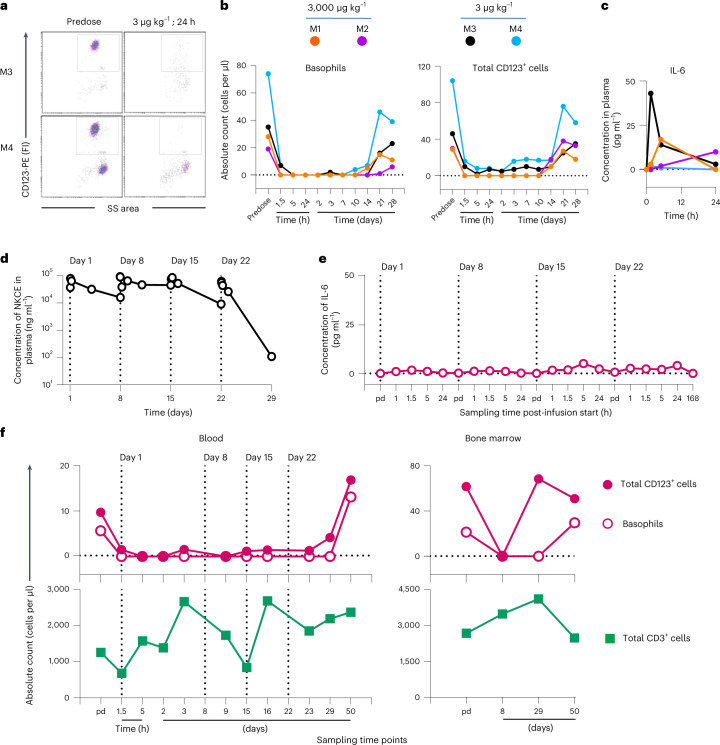
CD123-NKCE is safe and induces pharmacodynamic effects through the sustained depletion of CD123-positive cells in NHPs. **a**, Depletion of CD123-positive basophils (gated population) from the blood of monkeys M3 and M4 treated at the low dose of 3 µg kg^−1^ was analyzed by flow cytometry before dosing (predose, pd) and 24 h after the start of the infusion. **b**, Numbers of circulating CD123-positive basophils (left panel) and total CD123-positive leukocytes (right panel) at time of study in monkeys M1 (orange) and M2 (purple) treated with 3 mg kg^−1^, and monkeys M3 (black) and M4 (blue) treated with 3 µg kg^−1^. **c**, IL-6 concentration in plasma of monkeys M1, M2, M3 and M4 are shown before dosing (0), and 1.5, 5 and 24 h after the start of the treatment. **d**, Toxicokinetics of the CD123-NKCE molecule in male monkey M5 weekly treated at a dose of 3 mg kg^−1^ per administration for 4 weeks (on days 1, 8, 15 and 22). Plasma CD123-NKCE concentrations were determined before dosing (predose) and 1, 1.5, 5, 24 and 72 h after the start of the 1-h infusion on days 1, 8, 15 and before dosing and, 1, 1.5, 5, 24 and 168 h after the start of the last infusion on day 22. Values below the lower limit of quantification (LLOQ, 0.25 ng ml^−1^) are not reported on the graphs. Infusion days are indicated by vertical dotted lines. **e**, Plasma IL-6 concentrations of monkey M5 were monitored before dosing and 1, 1.5, 5 and 24 h after the start of the 1 h infusion on days 1, 8, 15 and before dosing and 1, 1.5, 5, 24 and 168 h after the start of the last fourth infusion on day 22. **f**, Upper panels show the number of circulating CD123-positive basophils (open symbols) and total CD123-positive leukocytes (closed symbols) in blood (left panel) or bone marrow (right panel), by timepoint in the study, for monkey M5, treated at a dose of 3 mg kg^−1^ per week. Lower panels show the number of circulating CD3-positive T cells (green square) in blood (left panel) or bone marrow (right panel).

**Extended Data Fig. 3:**
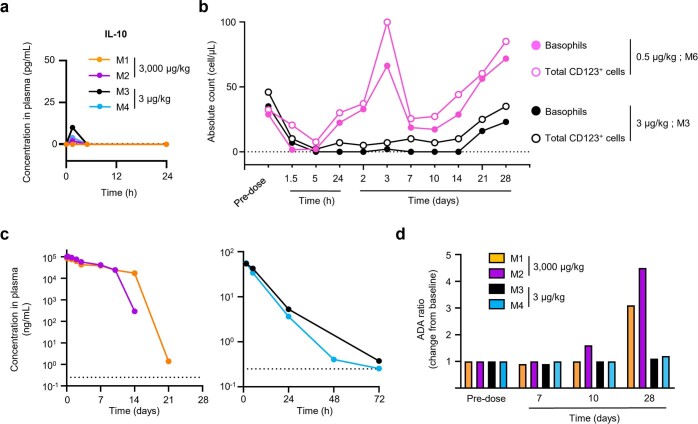
CD123-NKCE is safe and induces pharmacodynamic effects through the sustained depletion of CD123-positive cells in NHPs. **a**, Cytokine production in cynomolgus monkeys treated with the high and low doses of 3 mg/kg and 3 µg/kg as single 1-hour intravenous infusion, respectively. Plasma IL-10 concentrations are shown before dosing (0), and 1.5, 5 and 24 hours after the start of the treatment. **b**, Numbers of circulating CD123-positive basophils (close symbols) and total CD123-positive leukocytes (open symbols) at time of study in monkeys M6 (pink) and M3 (black) treated with 0.5 and 3 µg/kg as single 1-hour intravenous infusion, respectively. **c**, Pharmacokinetics of the CD123-NKCE molecule in monkeys M1 (orange) and M2 (purple) treated with 3 mg/kg (*left panel*), and monkeys M3 (black) and M4 (blue) treated with 3 µg/kg (*right panel*). Plasma CD123-NKCE concentrations were monitored 1.5, 5, 24, 48, 72, 168, 240, 336, 504 and 672 hours (that is 0.04, 0.06, 0.21, 1, 2, 3, 7, 10, 14, 21 and 28 days) after the start of the one-hour infusion. The lower limit of quantification (LLOQ; 0.25 ng/mL) is indicated by the horizontal dotted line. **d**, Individual Anti-Drug antibody (ADA) ratio of monkeys M1 (orange) and M2 (purple) treated with 3 mg/kg, and monkeys M3 (black) and M4 (blue) treated with 3 µg/kg. Presence of ADA in plasma was monitored at predose (baseline) and at day 1, 7, 10, and 28 after the start of the one-hour infusion.

We evaluated the pharmacokinetic/pharmacodynamics of CD123-NKCE administered by a single 1-hour i.v. infusion of a high (3 mg kg^−1^) or low (3 and 0.5 µg kg^−1^) doses in male cynomolgus monkeys (two animals each for the 3 mg kg^−1^ and 3 µg kg^−1^ doses and one animal for the 0.5 µg kg^−1^ dose). Treatment with CD123-NKCE promoted a sustained and complete depletion of CD123^+^ cells in the blood of all monkeys, for more than 10 days, at both the 3 mg kg^−1^ and 3 µg kg^−1^ doses (Fig. 6a,b), with only very small amounts (<50 pg ml^−1^) of the proinflammatory cytokines IL-6 and IL-10 released (Fig. 6c and Extended Data Fig. 3a) without any associated clinical signs.

A transient and partial depletion of CD123^+^ cells was observed in the monkey treated at the lowest dose (0.5 µg kg^−1^, Extended Data Fig. 3b), but 3 µg kg^−1^ was considered to be the lowest effective dose in this species. The pharmacokinetic profiles of the two monkeys treated at 3 mg kg^−1^ demonstrated a sharp drop in plasma concentrations from 7 to 10 days after dosing due to antidrug antibody (ADA) response (Extended Data Fig. 3c,d).

We further investigated the preclinical toxicology profile of CD123-NKCE, through an exploratory repeat-dose toxicity study in which eight monkeys (two per sex per dose) were treated weekly, for 4 weeks, at 3 or 0.1 mg kg^−1^ per administration. Exposure to CD123-NKCE lasted for at least 2 weeks at both doses (Extended Data Table 2), with the presence of ADA detected from the third administration (Day 15) in all monkeys except M5 (Fig. 6d and Extended Data Table 3). Transient minimal increases in IL-6 concentration were observed after each weekly administration, for both doses (Extended Data Table 4; maximum levels of 23 and 160 pg ml^−1^ for 0.1 and 3 mg kg^−1^ per administration, respectively). In particular, no substantial IL-6 release was observed in monkey M5 that did not exhibit an ADA response (Fig. 6e), and was exposed to CD123-NKCE throughout the study (Fig. 6d) with strong specific pharmacodynamics effects of CD123^+^ cell depletion in bone marrow and in blood up to 7 days after the last administration (Fig. 6f). In all the other animals, a sustained depletion of CD123-expressing cells was observed in the blood 1.5 h after the first administration and at least up to 24 h after the third administration. Moreover, all monkeys presented a complete depletion of CD123-positive cells from the bone marrow on day 9 (24 h after the second administration), for both doses (Extended Data Table 5), with a restoration of CD123-positive populations 1 week after the last administration.

No clinical signs, changes in body weight or body temperature and no effects on electrocardiogram potentially attributable to treatment with CD123-NKCE were observed, whatever the dose. Also, no compound-related adverse effects on hematological, coagulation, clinical chemistry or urinary parameters were observed. Overall, these results thus constitute proof-of-principle for the efficacy of CD123-NKCE in vivo, with no signs of toxicity.

## Discussion

**Extended Data Fig. 4:**
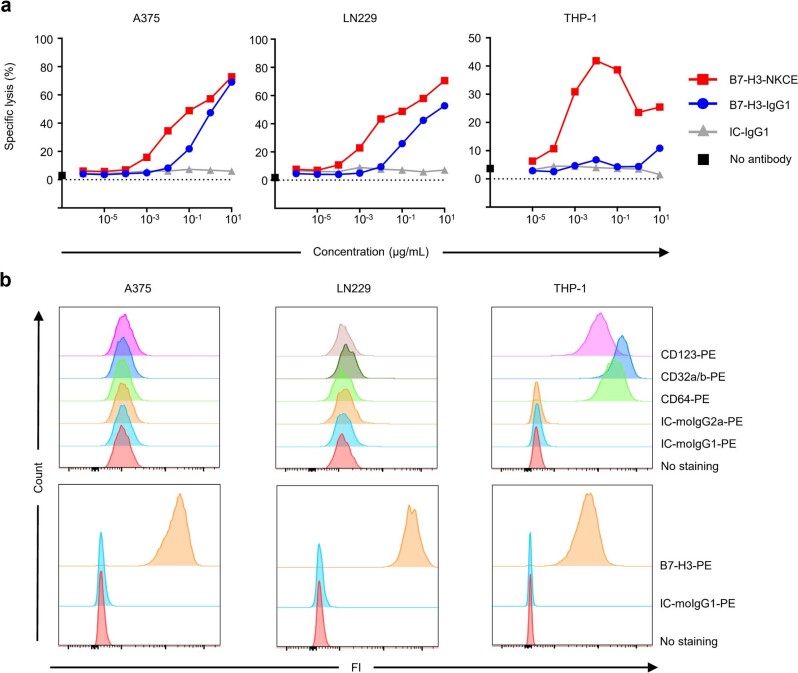
Expression of CD64 on target cells inhibits anti-B7-H3 antibody-mediated ADCC in vitro. **a**, Comparison of the cytotoxicities of an anti-B7-H3 antibody (B7-H3-IgG1) and of an anti-B7-H3 NKCE molecule co-engaging NKp46 and CD16a (B7-H3-NKCE). CD64^−^negative A375 and LN229 cells, and CD64-positive THP-1 cells were used as the targets and purified resting NK cells as effectors. The data shown are representative of three independent experiments. **b**, Phenotype of the target cells used in **a** showing the expression of B7-H3, CD32a/b, CD64 and CD123 by flow cytometry.

There is a clear unmet medical need for patients with AML who relapse after chemotherapy. The development of targeted therapies, such as monoclonal antibodies mediating ADCC has proved effective in clinical practice, particularly for the treatment of B cell leukemia^48^. Along the same lines, naked monoclonal antibodies have also been developed for the specific treatment of AML by targeting several antigens expressed on blasts, including, in particular, CD33 (refs. 49, 50), CD123 (refs. 12, 13) and, more anecdotally, CD135 (FLT-3) and CXCR4 (ref. 51). All these antibodies displayed some efficacy and functionality in preclinical studies and early clinical trials, but none was considered sufficiently potent in later phases of testing to become the standard of care for AML. In this study, we found that the expression of high-affinity FcγR CD64 on AML blasts interfered with the ADCC mediated by anti-CD123 antibodies. This observation was confirmed by results for other antigens (Extended Data Fig. 4a,b). Based on 3D-structure data for IgG1-Fc complexed with CD16a (ref. 52) and CD64 (ref. 53), showing that both molecules interact with overlapping binding sites on the Fc and given the three orders of magnitude higher affinity of binding to human IgG1 for CD64 than for CD16a (Extended Data Table 1), we hypothesized that the *cis* capture of antibody Fc at the blast cell surface by CD64 might prevent the binding of antibody Fc in *trans* to CD16a on NK cells, leading to the abolition of ADCC. The 3D-structures of IgG1-Fc complexed with CD32 (ref. 54) and the complement factor C1q (ref. 55) also revealed a highly probable competitive mode of binding to CD64, suggesting that CD64 expression may also protect cancer cells from antibody-dependent cell phagocytosis and complement-dependent cytotoxicity. A substantial proportion of patients with AML (about 30%) express CD64 on their blasts^38,39^. CD64 expression probably therefore protects cancer cells from ADCC, and potentially complement-dependent cytotoxicity and antibody-dependent cell phagocytosis in vivo, explaining the disappointingly low efficacy of antibodies acting via these modes of action in the treatment of patients with AML.

We report here the preclinical development of a new antibody-based NK cell engager technology, CD123-NKCE, targeting CD123 on malignant cells and coengaging CD16a and NKp46 on NK cells. We show that redirecting NK cells against cancer targets through binding to NKp46 circumvents the CD64-mediated inhibition of ADCC, with CD123-NKCE active and superior to Fc-engineered ADCC-enhancing IgG1 antibody targeting the same antigen, in vitro, ex vivo and in vivo, whatever the CD64 status of the target cells. Moreover, through their binding to NKp46, CD123-NKCE specifically target NK cells, a population of effector cells with promising perspectives for use in the treatment of cancer^15–18^, in terms of both efficacy and safety. The efficacy of CD123-NKCE to deplete CD123-positive cells ex vivo from human PBMCs and in vivo in NHP was not associated with the induction of strong cytokine release or clinical signs of toxicity. The activity, safety, pharmacokinetic and pharmacodynamics data provided here thus demonstrate the superiority of CD123-NKCEs over comparator cytotoxic antibodies in terms of antitumor activity, and their favorable safety profiles relative to T cell therapies for the treatment of AML.

## Methods

### NKCE expression and purification

The sequences encoding each polypeptide chain of the NKCE molecules were inserted into the pTT-5 vector between the *Hin*dIII and *Bam*HI restriction sites, as described previously^29^. The three vectors were used to cotransfect EXPI-293F cells (ThermoFisher Scientific, 100044202) in the presence of PEI (37 °C, 5% CO_2_, shaking at 150 rpm). Cells were seeded at a density of 10^6^ cells per ml in EXPI293 medium (Gibco, A1435101) supplemented with valproic acid (0.5 mM), glucose (4 g l^−1^) and tryptone N1 (0.5%), and cultured for 6 days. NKCE molecules were purified with rProtein A Sepharose Fast Flow resin (GE Healthcare, 17-1279-03), followed by cation ion exchange chromatography onto two HiTrap SP-HP 1 ml columns (GE Healthcare, 17-1151-01) in series and finally dialyzed overnight against 1× PBS. The CD123-NKCE batch used for the NHP study was produced at Sanofi in a 200 l bioreactor with a CHO stable-producer clone, and purified according to the Sanofi process development platform procedure.

### Recombinant protein cloning, production and purification

The human and cynomolgus recombinant proteins listed below were produced and purified at Innate Pharma as described previously^29^: human NKp46 (Gln22-Asn255, National Center for Biotechnology Information (NCBI) NM_004829.5), human neonatal Fc receptor (FcRn, NCBI P55899), human CD16a (human FcγRIIIA V and F isoforms, NCBI AAH36723), human CD32a (human FcγRIIA, NCBI AAH20823), human CD32b (human FcγRIIB, NCBI NP_003992), human CD16b (human FcγRIIIB, NCBI AAI28563), human CD64 (human FcγRI, NCBI P12314), cynomolgus NKp46 (Gln17-Asn254, NCBI NP_001271509.1), cynomolgus FcRn (NCBI Q8SPV9), cynomolgus CD16 (NCBI NP_001270121.1), cynomolgus CD32a (NCBI NP_001270598.1), cynomolgus CD32b (NCBI reference NP_001271060.1) and cynomolgus CD64 (NCBI AAL92095.1). The recombinant human CD123 was purchased from ACRO Biosystems (ILA-H52H6).

### Surface plasmon resonance study of binding

A Biacore T200 instrument (Cytiva, 28975001) was used with Series S CM5 sensor chips (Cytiva, 29149603) and experiments were performed at 25 °C.

Affinity capture of the NKCE sample was achieved with the human antibody capture kit (Cytiva, BR1008-39). Seven serial 1:1 dilutions of either human and cynomolgus NKp46 or human CD123 in HBS-EP + buffer (Cytiva, BR1006-69) were prepared at concentrations of ranging from 1.56 to 100 nM. The CD123-NKCE (0.06 µg ml^−1^) was captured on the anti-Fc chip at a flow rate of 10 µl min^−1^ for 90 s to yield maximal response (*R*_max_) values of approximately 30 RU. Proteins were injected for 240 s at a flow rate of 30 µl min^−1^ onto captured NKCE, followed by a dissociation phase of 1,200 s. All analyte concentrations were run in duplicate, together with multiple buffer blanks for double referencing. The capture surface was regenerated with regeneration solution (3 mol l^−1^ MgCl_2_) at a flow rate of 30 µl min^−1^ for 60 s. The data were evaluated with Biacore T200 Evaluation Software v.3.0 (Cytiva) using a 1:1 binding model with a mass transport limitation.

For FcR binding studies, CD123-NKCE molecules and control human IgG1 antibodies were immobilized (at about 700 RU) onto the dextran layer of a CM5 Series S sensor chip on flow cells 2 and 3 by amine coupling chemistry. Flow cell 1 activated with NHS/EDC alone and deactivated with ethanolamine served as a reference flow cell for online blank subtraction.

For all experiments other than the FcRn binding study, HBS-EP^+^ 1× was used as the running buffer. For the FcRn binding study, acetate buffer pH 5.6 replaced HBS-EP^+^. Serial dilutions of FcRn and FcγRs were sequentially injected over a period of 2 min, at a constant flow rate of 40 µl min^−1^ over the CM5 chip and allowed to dissociate for 10 min before regeneration (10 s of 10 mM NaOH 500 mM NaCl and 10 s of HBS-EP^+^ at a constant flow rate of 40 µl min^−1^ for FcγRs and FcRn, respectively).

The sensorgram sets of human and cynomolgus FcγRI were fitted with the 1:1 binding model. The sensorgram sets of human FcγRIIa, FcγRIIb, FcγRIIIaF, FcγRIIIaV and FcγRIIIb, and cynomolgus FcγRIIa, FcγRIIb and FcγRIII were fitted with a steady-state affinity model.

The sensorgram sets of human and cynomolgus FcRn were fitted with a two-state reaction model. The experiment was performed three times, on three different days, with the same Biacore CM5 chip. The affinities for human and cyno FcRn and FcγRI were calculated from the kinetic association (*k*_a_) and dissociation (*k*_d_) rate constants: dissociation constant (*K*_D_) = *k*_a_/*k*_d_.

Affinities for human and cynomolgus FcγRII and FcγRIII receptors were calculated from Scatchard plot fits.

### Biological samples

Healthy human buffy coats were provided by the Etablissement Français du Sang (EFS, the French blood service, Marseille; AC-2019-3428). Peripheral mononuclear cells (PBMC) were isolated from buffy coats by Ficoll density gradient centrifugation. Human NK cells were purified from PBMCs with a bead-based negative selection kit (Miltenyi, 130-092-657). Samples from patients with AML were provided by Institut Paoli-Calmettes (Marseille, SA-IPH-MImAbs Contract).

### Cell lines

KG-1a, Kasumi-6, GDM-1, MOLM-13 and THP-1 AML cell lines were purchased at ATCC. M-07e, EOL-1, Kasumi-1, F36-P, NB-4, OCI-AML2, MV4-11, OCI-AML3 and SKM-1 AML cell lines were purchased at DSMZ. Cells were cultured in Roswell Park Memorial Institute (RPMI)-1640 medium supplemented with 10% FBS, 2 mM l-glutamine, 1 mM sodium pyruvate and 1× nonessential amino acids (complete RPMI). Culture medium was supplemented with 25 mM HEPES for THP-1 cells. Quantification of CD123 expression on AML cell lines (antibody binding capacity) was performed by flow cytometry using mouse IgG calibrator kit (BioCytex, CP051). Anti-CD123 antibody 9F5 and isotype control (BD Biosciences, 555642 and 553447) were used at saturating concentration (10 µg ml^−1^) for the quantification. CD64 and CD32a/b expression in THP-1 cells was silenced with CRISPR–Cas9 endonucleases. For the generation of CD64-deficient THP-1 cells (THP-1 CD64-KO), 2.5 × 10^6^ cells were nucleofected (Neon Transfection System, 100 µl tip, 1,700 V, 20 ms, one pulse) with two sgRNAs (CD64.1: CUUGAGGUGUCAUGCGUGGA; CD64.2: AAGCAUCGCUACACAUCAGC; Synthego) at a Cas9:sgRNA ratio of 1:9 (Alt-R S.p. Cas9 Nuclease 3NLS, Integrated DNA Technology). For the generation of CD32-deficient THP-1 cells (THP-1 CD32-KO), 2.5 × 10^6^ cells were nucleofected with two sgRNAs (CD32A, AUGUAUGUCCCAGAAACCUG; CD32B, AAGCAUAUGACCCCAAGGCU; Integrated DNA Technologies) at a CAS9:sgRNA ratio of 1:9. Absence of CD64 and CD32 expression was confirmed by flow cytometry and cells were either sorted or subcloned.

### NK cell-based cytotoxic assay

For cytotoxic assays performed on AML cell lines (that is, KG-1a, M-07e, EOL-1, Kasumi-1, F36-P, Kasumi-6, GDM-1, NB-4, OCI-AML2, MV4-11, OCI-AML3, SKM-1, MOLM-13, THP-1 or THP-1 CD64-KO, THP-1 CD32-KO), target cells were loaded with Cr-51.

Seven primary samples from patients with AML collected at diagnosis were used for the study. They were composed by PBMC and AML blasts. The day before the cytotoxic assay, primary samples were thawed, cells were counted with a trypan blue exclusion test and cultured in complete RPMI at 2 × 10^6^ cells per ml. The viability of primary AML cells was monitored at each step of the experimental process. Primary cells AML samples were loaded with CalceinAM (8 µg ml^−1^; Life Technologies, C3100MP) for 30 min in the presence of 2.5 mM probenecid (ThermoFisher, P36400). Dilution ranges of both test and control items from 5 to 2.10^−5^ µg ml^−1^ (1/12 serial dilution) and 5 to 5.10^−7^ µg ml^−1^ (1/10 serial dilution) were performed for experiments with primary AML cells or AML cell lines, respectively.

Antibodies, target cells (roughly 3,000 cells) and human NK cells (roughly 30,000 cells) were successively added to each well of round-bottomed 96-well plates. After 4 h of coincubation, the supernatant was transferred to a Lumaplate (for Cr-51) or a flat-bottomed culture plate (for CalceinAM).

Cr-51 released from dead target cells was determined with a TopCount NXT (Microplate Scintillation and Luminescence Counter; Perkin Elmer). Radioactivity was measured by counting γ-emission for 60 s for each well. The results are expressed in cpm (counts per minute). CalceinAM released by dead target cells was determined by measuring the number of relative fluorescence units (RFU) with a luminometer (EnSpire Multimode Plate Reader, Perkin Elmer; excitation at *λ* = 495 nm and emission at *λ* = 516 nm). The percentage specific lysis was calculated with the following formula:

Specific lysis (%) = (ER (cpm or RFU) − SR (cpm or RFU))/(MR (cpm or RFU) − SR (cpm or RFU)) × 100 where ER = experimental release, SR = spontaneous release and MR = maximal release.

EC_50_ were determined by fitting the data with nonlinear regression curve model (log(agonist) versus response–variable slope (four parameters)) with GraphPad Prism Software v.8.0.2.

### NK cell degranulation assay with AML samples

Tested items and PBMCs from patients with AML were added to each well of round-bottomed 96-well plates. After overnight coincubation with the NKCE molecules or antibodies, antihuman CD107a and CD107b antibodies (Miltenyi, 130-111-621 and 130-118-818) were added for 4 h. Cells were then washed and stained with the following mixture: viability markers, anti-CD45 (Miltenyi, 130-110-771), anti-CD33 (BD Biosciences, 564588), anti-CD56 (BD Biosciences, 557747) and anti-CD3 (BD Biosciences, 740187) antibodies. Cells were then washed, fixed and analyzed by flow cytometry. The data obtained were analyzed with Flowjo Software to assess NK cell degranulation by monitoring the expression of CD107a/b on NK cells identified as living CD45^+^CD33^−^CD56^+^CD3^−^ cells.

### NK cell activation assay with AML cell lines

A dilution range from 15 to 15.10^−7^ µg ml^−1^ (1/10e serial dilution) was performed for both test and control items. The tested items, MOLM-13 cells (roughly 50,000 cells) and human NK cells (roughly 50,000 cells) from healthy donors were successively added to each well of round-bottomed 96-well plates. Control conditions were performed by adding only 50,000 resting NK cells by well. BD GolgiSTOP solution (BD Biosciences, 554724) was added at a final dilution of 1/6,000 in each well. A positive control of NK cell activation was performed by using Phorbol 12-myristate 13-acetate (PMA, 125 ng ml^−1^ final; SIGMA, P8139) and of Ionomycin (IONO, 1 µg ml^−1^ final; SIGMA, I0634) added on 50,000 NK cells. Each condition was performed in simplicate. After 4 h of coincubation at 37 ± 1 °C and 5 ± 1% CO_2_, an extracellular staining was performed for CD3, CD56, CD107a and CD107b (Human NK cell activation panel cocktail; Miltenyi, 130-095-212) and CD69 (Miltenyi, 130-113-523). An intracellular staining was performed for IFN-γ (Biolegend, 502536), TNF-α (BD Biosciences, 563996) and MIP-1β (BD Biosciences, 550078). Cells were analyzed by flow cytometry (Supplementary Fig. 3). Parameters were recorded with BD FACSDiva v.8.0 software and the analyses were done with FlowJo v10.5.2 software. Analysis of the percentage of NK cell activation was done with GraphPad prism v.8.0.2.

### In vitro pharmacodynamic assessment and cytokine release in human PBMC

PBMCs from human healthy donors (*n* = 10) were seeded in 190 µl complete culture medium (500,000 cells per well) in 96-well U-bottomed plates (Costar, Ultra low binding CLS7007), and incubated at 37 °C and 5% CO_2_ for 20 h in presence of serial dilutions of CD123-NKCE, IC-NKCE control or CD123-TCE molecules. The basophil population, defined as TCRαβ^-^CD14^-^IgE^+^ viable cells, was analyzed by flow cytometry and the absolute concentrations of cytokines released into the supernatant were analyzed by mesoscale discovery (MSD) assay.

For flow cytometry analysis, cell pellets were suspended in cold 50 µl staining buffer (Miltenyi, AutoMACS Running Buffer 130-091-221) supplemented with 1 µl of human FcR blocking reagent (Miltenyi, 130-059-901). A mixture of PBMC subset-specific antibodies and the viability reagent were added to the PBMC suspension according to the supplier’s instructions. As a fluorescence minus one control, additional points were obtained by labeling PBMC with the same mixture, but with each labeling antibody replaced in turn by its corresponding isotype control. Cells and antibody mixtures were incubated for 1 h at 4 °C in the dark, and then washed twice with 200 µl of staining buffer by centrifugation at 300*g* for 5 min at 4 °C. Cells were analyzed with a MACSQuant Analyzer from Miltenyi Biotec. Raw data were analyzed with VenturiOne v.6.1 software (Applied Cytometry Inc.). The gating strategy can be found in Supplementary Fig. 4.

For MSD assay, cell supernatant was diluted in MSD buffer following the manufacturer’s instructions. Diluted samples or prediluted multi-analyte calibrator samples were added to the precoated plate supplied in the kit. A solution of detection antibodies conjugated to electrochemiluminescent labels (MSD SULFO-TAG) was added and the plates were incubated at room temperature for 2 h before measurements. Data were analyzed with Excel 2019 software. The concentrations of IL-6, IL-1β, IFN-γ and TNF-α were determined from electrochemiluminescent signals by back-fitting to a calibration curve established with a four-parameter logistic model with 1/Y2 weighting.

### Animal care

All animal procedures were approved by the Sanofi Animal Care and Use Committee, followed the French and European regulations on care and protection of the Laboratory Animals, and in accordance with the standards of the Association for Assessment and Accreditation of Laboratory Animal Care (AAALAC).

### Antitumor activity against human MOLM-13 AML cells injected into severe combined immunodeficiency mice

The activity of the surrogate CD123-NKCE was evaluated in a disseminated human AML model consisting in MOLM-13 cells implanted in the tail vein of female severe combined immunodeficiency mice on day 0. Control groups were left untreated. Graph presented are the pooled results of four independent experiments (*n* = 20 mice per treatment group and 40 mice in the control group). Surrogate CD123-NKCE and anti-CD123 antibody were administered at doses of 5, 0.5 and 0.25 mg kg^−1^ by intraperitoneal injections on day 1.

Mice were checked and adverse clinical reactions noted. Individual mice were weighed daily until the end of the experiment (day 70). Mice were euthanized when they were considered moribund according to predefined criteria, to prevent animal suffering. The disease-related clinical signs considered critical were limb paralysis, ascites, palpable internal tumor masses, morbidity or a loss of at least 20% of total body weight loss.

For NK cell depletion, 100 µl of polyclonal anti-asialo-GM1 (Poly21460, Biolegend) antibody was injected intraperioneally into recipient mice at the indicated time points.

### Pharmacodynamic activity in NHPs

A qualified flow cytometry panel composed of antibodies against the antigens CD45 (BD Biosciences ref. no. 563530, Clone D058-1283), CD14 (Miltenyi Biotec ref. no. 130-110-518, clone REA599), CD203c (Invitrogen ref. no. 17–2039, clone NP4D6), CD193 (Biolegend ref. no. 310708, clone 5E8), IgE (Miltenyi Biotec ref. no. 130-117-931, clone REA1049), CD123 (BD Biosciences ref. no. 554529, clone 7G3), CD33 (Miltenyi Biotec ref. no. 130-113-350, clone AC104.3E3) and the viability marker Zombie Nir (Biolegend ref. no. 423106) was used to evaluate the phenotype and counts of basophils and total CD123-positive immune cells in cynomolgus monkey blood and bone marrow samples. Blood (100 µl) and bone marrow samples (50 µl) were collected into a K3-EDTA anticoagulation air-vacuum tubes, incubated with a lysis solution (Biocytex CP025) for 10 min and centrifuged at 300*g* at room temperature for 5 min with Dulbecco’s PBS (Sigma D8537) before staining for 10 min, washing and fixation. We added 100 µl of flow count beads (Beckman ref. no. A91346) to the sample before acquisition on a Beckman Coulter Gallios (single dose pharmacokinetic/pharmacodynamics study) and BD FACS Verse (repeated dose toxicity study) instruments. The gating strategy for CD123-positive immune cells can be found in Supplementary Fig. 5.

### Cytokine determinations on NHP plasma

In the single dose NHP pharmacokinetic/pharmacodynamics study, a qualified electrochemiluminescence assay method was developed using the MSD V-PLEX Proinflammatory Panel NHP kit (K15056D) for the quantification of IL-2, IFN-γ, IL-6 and IL-10 in monkey K3-EDTA plasma. In the repeated dose NHP toxicity study, an exploratory electrochemiluminescence assay method was developed using the MSD U-PLEX Proinflammatory Combo1 NHP kit (K150070K-2) for the quantification of IL-6, IL-2, IL-10, TNF-α, IFN-γ, IL-1β and IL-8 in monkey K3-EDTA plasma. Samples were analyzed according to the manufacturer’s recommendations. Analyses were performed in duplicate.

### Pharmacokinetic/pharmacodynamic and toxicology studies in NHPs

CD123-NKCE solutions for administration (at concentrations of 0.1, 0.6, 20 and 600 µg ml^−1^) were prepared extemporaneously by diluting the stock solution in vehicle. They were kept at room temperature before and during administration. We used polypropylene, polycarbonate or PETG containers for dilutions, to prevent adsorption. These containers were coated with 100 ppm PS80 in 0.9% NaCl before use. The tubing used for each i.v. administration (syringe/winged needle) was coated, by successive flushes, with a solution of 100 ppm PS80 in 0.9% NaCl. The dosing volume was 5 ml kg^−1^ by 1 h i.v. infusion.

In the single dose pharmacokinetic/pharmacodynamics study, two males per group administered 3 or 3,000 µg kg^−1^ and one male administered 0.5 µg kg^−1^.

In the exploratory repeat-dose toxicity study, two animals per sex and per dose administered 0.1 or 3 mg kg^−1^ per administration, once weekly, for 4 weeks (on days 1, 8, 15 and 22). One monkey per sex per dose was euthanized and necropsied 1 week after the last administration and the remaining monkeys were euthanized and necropsied at 4 weeks after the last administration. The parameters evaluated included mortality, clinical signs, body weight, injection site examination, body temperature, electrocardiography parameters, hematology, clinical chemistry, coagulation and urinalysis, macroscopic observations, organ weights and histopathologic findings.

In both studies, serial blood samples were withdrawn from the brachial or saphenous or cephalic vein into K3-EDTA polypropylene tubes for plasma CD123-NKCE concentrations, 1.5, 5, 24, 48, 72, 168, 240, 336, 504 and 672 h after the start of the infusion, for the single dose pharmacokinetic/pharmacodynamics study and predose, 1, 1.5, 5, 24, 72 and 168 h after the start of each weekly infusion for the repeated dose toxicology study. Blood samples were placed on wet ice and centrifuged. The plasma samples obtained were frozen at −80 °C until analysis.

CD123-NKCE concentrations in plasma were determined by a dedicated immunoassay method in which CD123-NKCE were captured by biotin-coupled CD123 recombinant proteins and detected with a monkey-adsorbed Alexa Fluor-conjugated-goat antihuman IgG, with a lower limit of quantification at 0.250 ng ml^−1^.

### Quantification and statistical analysis

Detailed information concerning the statistical methods used is provided in the figure legends. Statistical analyses were performed with GraphPad Prism software v.8.0.2, and v.8.3.0. Kaplan–Meier methods were used for survival analysis. When sample size was sufficiently large, the normality of populations was assessed with the d’Agostino-Pearson omnibus normality test. If the data were not normally distributed, the statistical significance of differences between paired sample populations was determined with the two-sided Wilcoxon matched-pair signed rank test. *n* is the number of samples used in the experiments. The means or medians are shown, with or without error bars indicating the s.d. Significance is indicated as follows: *P ≤ 0.05; **P ≤ 0.01; ***P ≤ 0.001, ****P ≤ 0.0001. Four-parameter nonlinear regression analysis was used to calculate the CD123-NKCE EC_50_.

### Reporting summary

Further information on research design is available in the Nature Portfolio Reporting Summary linked to this article.

## Online content

Any methods, additional references, Nature Portfolio reporting summaries, source data, extended data, supplementary information, acknowledgements, peer review information; details of author contributions and competing interests; and statements of data and code availability are available at 10.1038/s41587-022-01626-2.

### Supplementary information

Supplementary Information Supplementary Figs. 1–5.

Reporting Summary

## Figures and Tables

**Extended Data Table 1:**
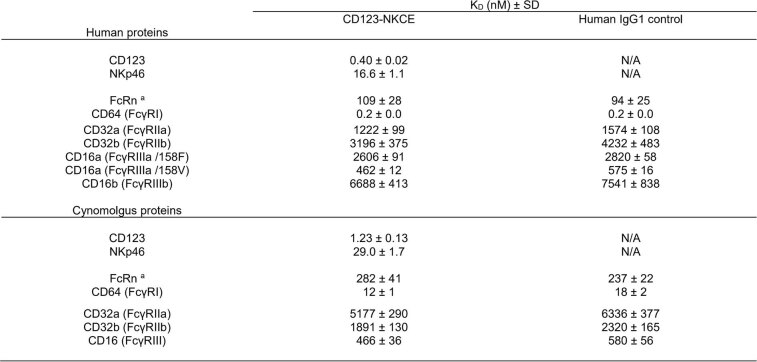
Monovalent affinities of CD123-NKCE binding to human and cynomolgus ligands

^a^experiment performed at +25 °C pH5.6; default condition: +25 °C pH7.4. N/A: not applicable. SD: standard deviation. K_D_: Dissociation constant. N = 5 for CD123 and NKp46 and N = 3 for all other proteins.

**Extended Data Table 2:**
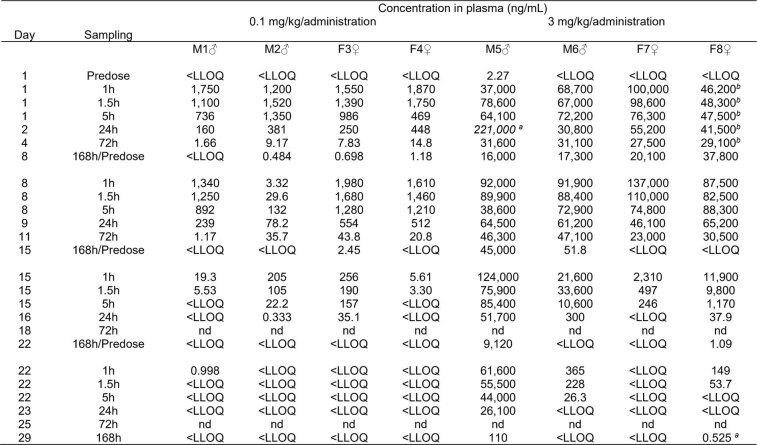
Individual CD123-NKCE plasma concentration values of cynomolgus monkeys treated with CD123-NKCE

Individual CD123-NKCE plasma concentration values after a weekly repeat 1-hour intravenous infusion at 0.1 and 3 mg/kg/administration for 4 weeks (on Days 1, 8, 15 and 22) to cynomolgus monkeys are shown. Values are rounded to 3 significant figures. LLOQ (Lower Limit Of Quantification) 0.250 ng/mL;

^a^aberrant value excluded for TK analysis;

^b^Given as indicative due to a technical issue during 1-hour infusion on Day 1 (that is, 50% of the dose received subcutaneously); nd: not done

**Extended Data Table 3:**
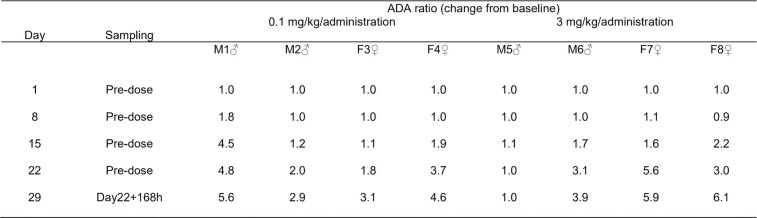
Individual anti-drug antibody (ADA) ratio of cynomolgus monkeys treated with CD123-NKCE

Individual anti-drug antibody (ADA) ratio (fold-change from baseline value collected at Day 1 Predose) after a weekly repeat 1-hour intravenous infusion of CD123-NKCE at 0.1 and 3 mg/kg/administration for 4 weeks (on Days 1, 8, 15 and 22) to cynomolgus monkeys are shown. Under the assay conditions, the presence of ADA is considered to be significant when the ADA ratio is above 3.0 or, for ratio below 3.0, when the ratio increase with time.

**Extended Data Table 4:**
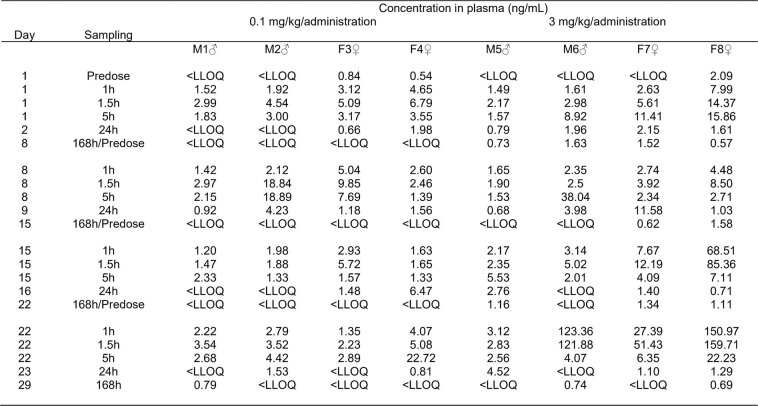
Individual IL-6 plasma concentration values of cynomolgus monkeys treated with CD123-NKCE

Individual IL-6 plasma concentration values after a weekly repeat 1-hour intravenous infusion of CD123-NKCE at 0.1 and 3 mg/kg/administration for 4 weeks (on Days 1, 8, 15 and 22) to cynomolgus monkeys are shown. LLOQ (Lower Limit Of Quantification): 0.53 pg/mL.

**Extended Data Table 5:**
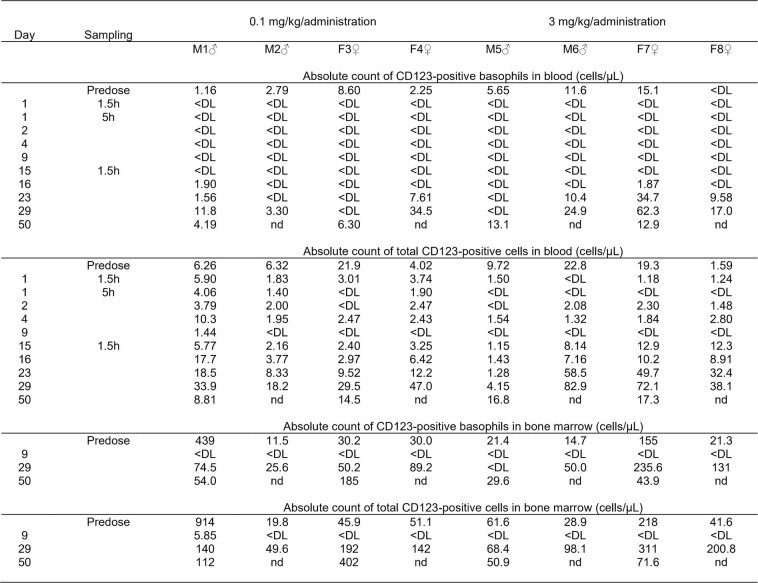
Individual absolute counts of basophils and total CD123-positive cells in blood and bone marrow of cynomolgus monkeys treated with CD123-NKCE

Individual absolute counts of basophils and total CD123-positive cells in blood and bone marrow after a weekly repeat 1-hour intravenous infusion of CD123-NKCE at 0.1 and 3 mg/kg/administration for 4 weeks to cynomolgus monkeys are shown. DL (detection limit):1.15 cells/µL (blood) or 2.10 cells/µL (bone marrow); nd: not done.

## Data Availability

All data supporting the results are available in the main text or the supplementary materials. The detailed molecular organization and the sequences of the NKCE used in the present study can be found in Supplementary Figs. 1 and 2, and in patents WO2016207273 and WO2022144836A1. Recombinant proteins were built from sequences found at https://www.ncbi.nlm.nih.gov.
