# Supplementary material for: Control of acute myeloid leukemia by a trifunctional NKp46-CD16a-NK cell engager targeting CD123
Source: Nat Biotechnol. 2023 Jan 12;41(9):1296–306. doi: 10.1038/s41587-022-01626-2 (PMC10497414; doi:10.1038/s41587-022-01626-2)

nature biotechnology

Article

https://doi.org/10.1038/s41587-022-01626-2

Control of acute myeloid leukemia by a trifunctional NKp46-CD16a-NK cell engager targeting CD123

In the format provided by the authors and unedited

Supplementary information Figure 1

Molecular design and amino acid sequences of the NK cell engager molecules

- <u>CD123-NKCE :</u> NKp46-Fc-CD123

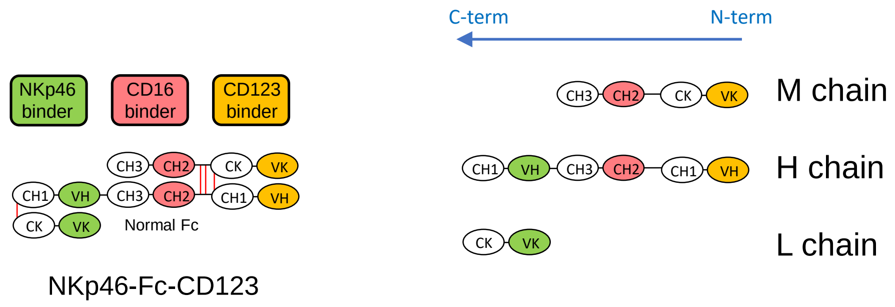

NKCE trifunctional format is constituted by the assembly of three polypeptide chains, namely chain M, H, and L, with respectively the following domain arrangement (ABD_L)1-CK-H-CH2-CH3, (ABD_H)1-CH1-H-CH2-CH3-(ABD_H)2-CH1, and (ABD_L)1-CK. The antigen binding domains (ABD_H)1 and (ABD_L)1 are the antibody heavy and light chain variable domains that bind to the target antigen on cancer cells (orange). (ABD_H)2 and (ABD_L)2 are the antibody heavy and light chain variable domains that bind to NKp46 on NK cells (green). CH1, CH2, CH3 and H are the human IgG1 constant domains and hinge. CK is the human kappa light chain constant domain. (Patent N° WO2016207273 and WO2022144836A1)

Sequences of mature proteins:

- M chain:

DIVMTQSPDSLAVSLGERATINCESSQSLLSSGNQKNYLTWYQQKPGQPPKPLIYWASTRESGVPDRFSGSGSGTDFTLTISSLQAEDVAVYYCQNDYSYPYTFGQGTKLEIKRTVAAPSVFIFPP
SDEQLKSGTASVVCLLNNFYPREAKVQWKVDNALQSGNSQESVTEQDSKDSTYSLSSTLTLSKADYEKHKVYACEVTHQGLSSPVTKSFNRGECDKTHTCPPCPAPELLGGPSVFLFPPKPKDTLM
ISRTPEVTCVVVDVSHEDPEVKFNWYVDGVEVHNAKTKPREEQYNSTYRVVSVLTVLHQDWLNGKEYKCKVSNKALPAPIEKTISKAKGQPREPQVYTLPPSREEMTKNQVSLTCLVKGFYPSDIA
VEWESNGQPENNYKTTPPVLDSDGSFFLYSKLTVDKSRWQQGNVFSCSVMHEALHNHYTQKSLSLSPGK

- H chain:

EVQLVQSGAEVKKPGESLKISCKGSGYSFTDYYMKWARQMPGKGLEWMGDIIPSSGATFYNQKFKGQVTISADKSISTTYLQWSSLKASDTAMYYCARSHLLRASWFAYWGQGTMVTVSSASTKGP
SVFPLAPSSKSTSGGTAALGCLVKDYFPEPVTVSWNSGALTSGVHTFPAVLQSSGLYSLSSVVTVPSSSLGTQTYICNVNHKPSNTKVDKRVEPKSCDKTHTCPPCPAPELLGGPSVFLFPPKPKD
TLMISRTPEVTCVVVDVSHEDPEVKFNWYVDGVEVHNAKTKPREEQYNSTYRVVSVLTVLHQDWLNGKEYKCKVSNKALPAPIEKTISKAKGQPREPQVYTLPPSREEMTKNQVSLTCLVKGFYPS
DIAVEWESNGQPENNYKTTPPVLDSDGSFFLYSKLTVDKSRWQQGNVFSCSVMHEALHNHYTQKSLSLSPGSTGSQVQLVQSGAEVKKPGSSVKVSCKASGYTFSDYVINWVRQAPGQGLEWMGEI
YPGSGTNYYNEKFKAKATITADKSTSTAYMELSSLRSEDTAVYYCARRGRYGLYAMDYWGQGTTVTVSSASTKGPSVFPLAPSSKSTSGGTAALGCLVKDYFPEPVTVSWNSGALTSGVHTFPAVL
QSSGLYSLSSVVTVPSSSLGTQTYICNVNHKPSNTKVDKRVEPKSCDKTHS

- L chain:

DIQMTQSPSSLSASVGDRVTITCRASQDISNYLNWYQQKPGKAPKLLIYYTSRLHSGVPSRFSGSGSGTDFTFTISSLQPEDIATYFCQQGNTRPWTFGGGTKVEIKRTVAAPSVFIFPPSDEQLK
SGTASVVCLLNNFYPREAKVQWKVDNALQSGNSQESVTEQDSKDSTYSLSSTLTLSKADYEKHKVYACEVTHQGLSSPVTKSFNRGEC

Supplementary information Figure 2

Molecular design and amino acid sequences of the NK cell engager molecules

- CD123-NKCE : NKp46-Fc null-CD123

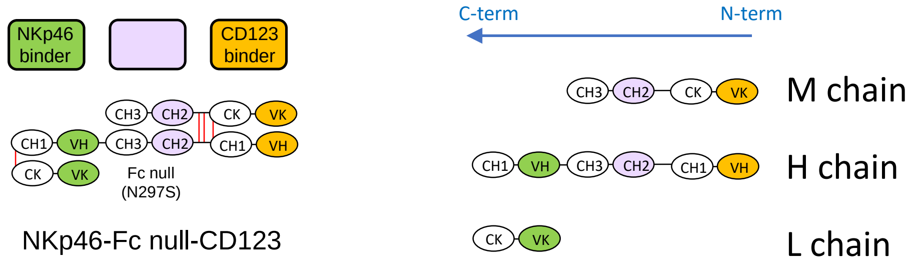

NKp46-Fc null-CD123

NKCE bifunctional format is constituted by the assembly of three polypeptide chains, namely chain M, H, and L, with respectively the following domain arrangement (ABD_L)1-CK-H-CH2-CH3, (ABD_H)1-CH1-H-CH2-CH3-(ABD_H)2-CH1, and (ABD_L)1-CK. The antigen binding domains (ABD_H)1 and (ABD_L)1 are the antibody heavy and light chain variable domains that bind to the target antigen on cancer cells (orange). (ABD_H)2 and (ABD_L)2 are the antibody heavy and light chain variable domains that bind to NKp46 on NK cells (green). CH1, CH2, CH3 and H are the human IgG1 constant domains and hinge. CK is the human kappa light chain constant domain. CH2 domain (pink) was mutated at position 297 (N297S) to inhibit binding to FcγRs (Fc null).
(Patent N° WO2016207273 and WO2022144836A1)

Sequences of mature proteins:

- M chain:

DIVMTQSPDSLAVSLGERATINCESSQSLLSSGNQKNYLTWYQQKPGQPPKPLIYWASTRESGVPDRFSGSGSGTDFTLTISSLQAEDVAVYYCQNDYSYPYTFGQGTKLEIKRTVAAPSVFIFPP
SDEQLKSGTASVVCLLNNFYPREAKVQWKVDNALQSGNSQESVTEQDSKDSTYSLSSTLTLSKADYEKHKVYACEVTHQGLSSPVTKSFNRGECDKTHTCPPCPAPELLGGPSVFLFPPKPKDTIM
ISRTPEVTCVVVDVSHEDPEVKFNWYVDGVEVHNAKTKPREEQY**S**STYRVVSVLTVLHQDWLNGKEYKCKVSNKALPAPIEKTISKAKGQPREPQVYTLPPSREEMTKNQVSLTCLVKGFYPSDIA
VEWESNGQPENNYKTTPPVLDSDGSFFLYSKLTVDKSRWQQGNVFSCSVMHEALHNHYTQKSLSLSPGK

- H chain:

EVQLVQSGAEVKKPGESLKISCKGSGYSFTDYYMKWARQMPGKGLEWMGDIIPSSGATFYNQKFKGQVTISADKSISTTYLQWSSLKASDTAMYYCARSHLLRASWFAYWGQGTMVTVSSASTKGP
SVFPLAPSSKSTSGGTAALGCLVKDYFPEPVTVSWNSGALTSGVHTFPAVLQSSGLYSLSSVVTVPSSSLGTQTYICNVNHKPSNTKVDKRVEPKSCDKTHTCPPCPAPELLGGPSVFLFPPKPKD
TLMISRTPEVTCVVVDVSHEDPEVKFNWYVDGVEVHNAKTKPREEQY**S**STYRVVSVLTVLHQDWLNGKEYKCKVSNKALPAPIEKTISKAKGQPREPQVYTLPPSREEMTKNQVSLTCLVKGFYPS
DIAVEWESNGQPENNYKTTPPVLDSDGSFFLYSKLTVDKSRWQQGNVFSCSVMHEALHNHYTQKSLSLSPGSTGSQVQLVQSGAEVKKPGSSVKVSCKASGYTFSDYVINWVRQAPGQGLEWMGEI
YPGSGTNYYNEKFKAKATITADKSTSTAYMELSSLRSEDTAVYYCARRGRYGLYAMDYWGQGTTVTVSSASTKGPSVFPLAPSSKSTSGGTAALGCLVKDYFPEPVTVSWNSGALTSGVHTFPAVL
QSSGLYSLSSVVTVPSSSLGTQTYICNVNHKPSNTKVDKRVEPKSCDKTHS

- L chain:

DIQMTQSPSSLSASVGDRVTITCRASQDISNYLNWYQQKPGKAPKLLIYYTSRLHSGVPSRFSGSGSGTDFTFTISSLQPEDIATYFCQQGNTRPWTFGGGTKVEIKRTVAAPSVFIFPPSDEQLK
SGTASVVCLLNNFYPREAKVQWKVDNALQSGNSQESVTEQDSKDSTYSLSSTLTLSKADYEKHKVYACEVTHQGLSSPVTKSFNRGEC

N297S mutation is shown in bold font

Supplementary information Figure 3

NK-cell activation assay with MOLM-13 AML cell line / NK-cell gating strategy and analysis

<u>NK cells:</u> Time Gate / Single cells (SSC-A SSC-W) / Living cells (livedead negative) / Leucocytes ($CD45^{+}$)/ $CD3^{-}$ / $CD56^{+}$

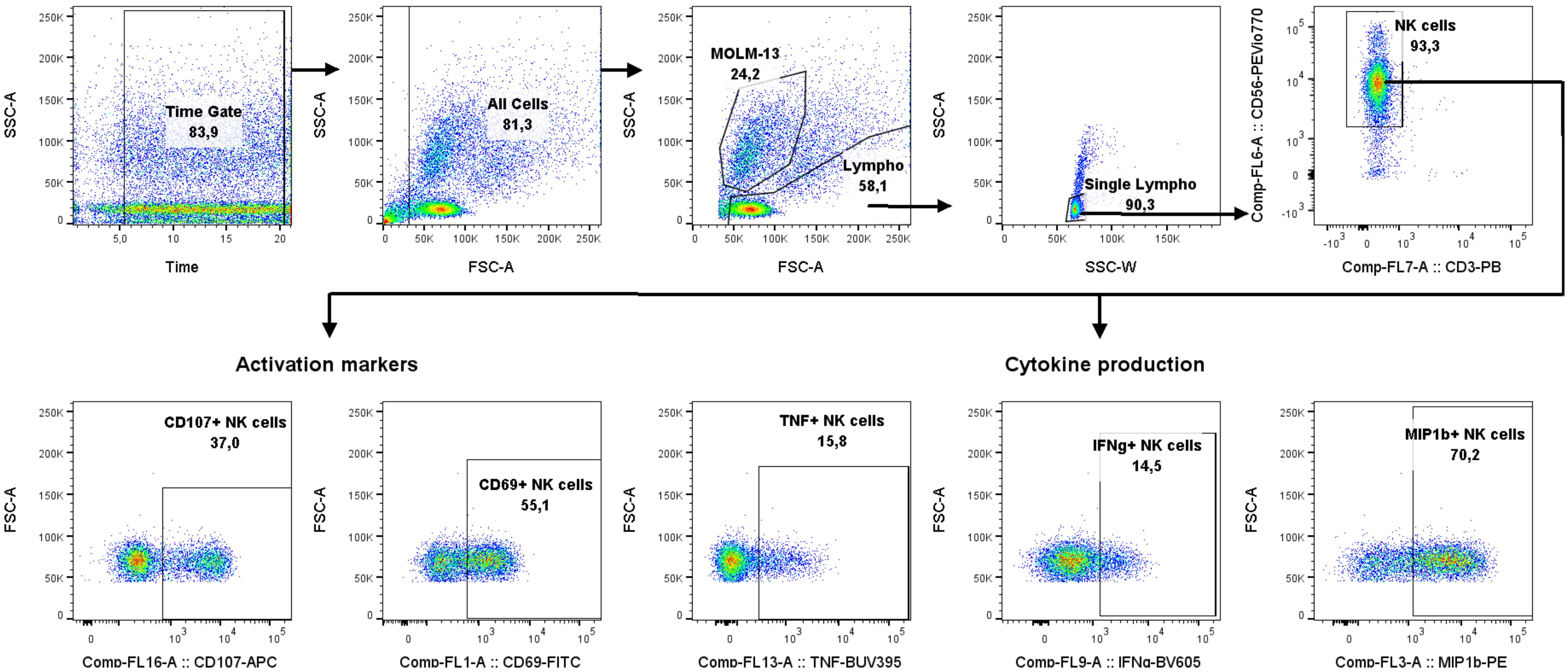

Results for NK cells + MOLM-13 treated with CD123-NKCE at 1.5 µg/mL are shown for one healthy donor

Supplementary information Figure 4
Depletion of CD123^{+} normal immune cells in Healthy Donor PBMCs treated overnight with CD123-NKCE

Gating strategy for basophils in human PBMC

Basophil gating strategy:
- FSC/SSC ; single cells; viable cells
- TCR^{-} / HLA-DR^{-} CD14^{-} / IgE^{+}(/CD203c^{+})

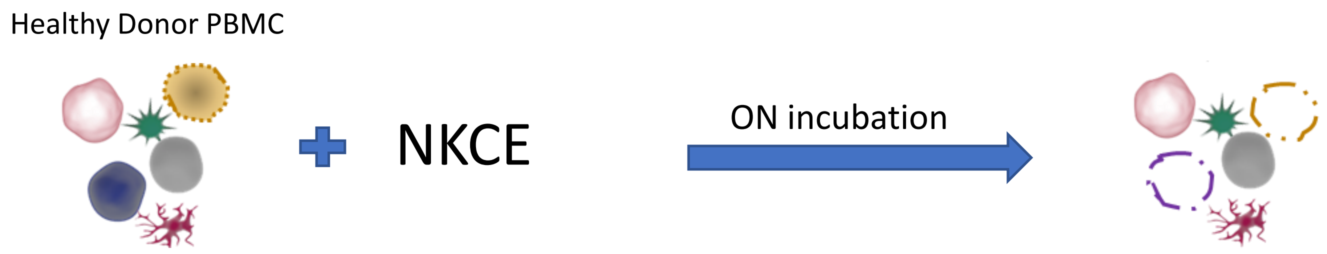

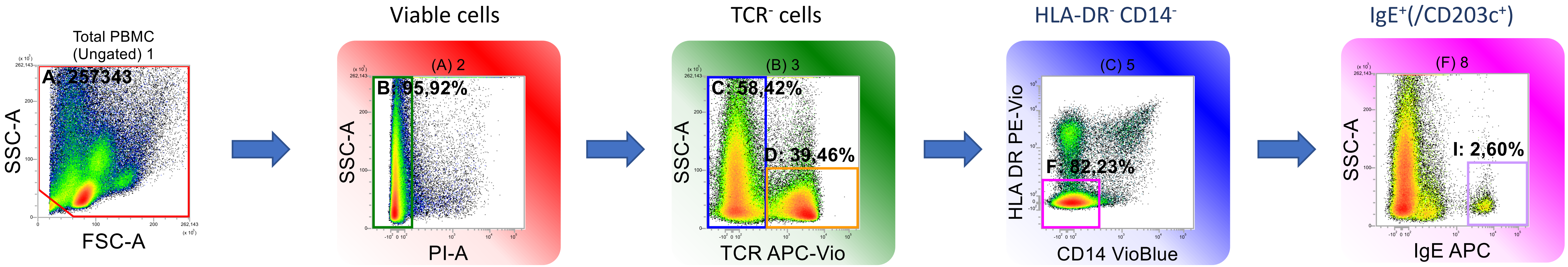

Untreated control is shown

Supplementary information Figure 5
Basophil and CD123-positive cell gating strategy in non human primate

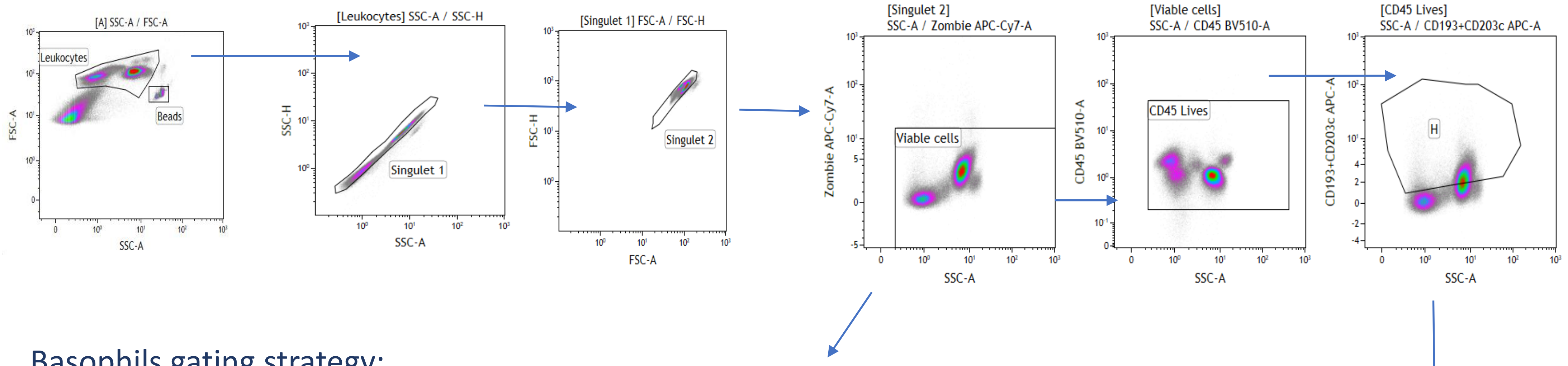

Basophils gating strategy:

- FSC/SSC ; single cells; viable cells
- CD45^{+}
- CD193^{+}/CD203c^{+}
- IgE^{+} (i.e. FcεRI^{+})

Total CD123^{+} cells gating strategy:

- FSC/SSC ; single cells; viable cells
- CD123

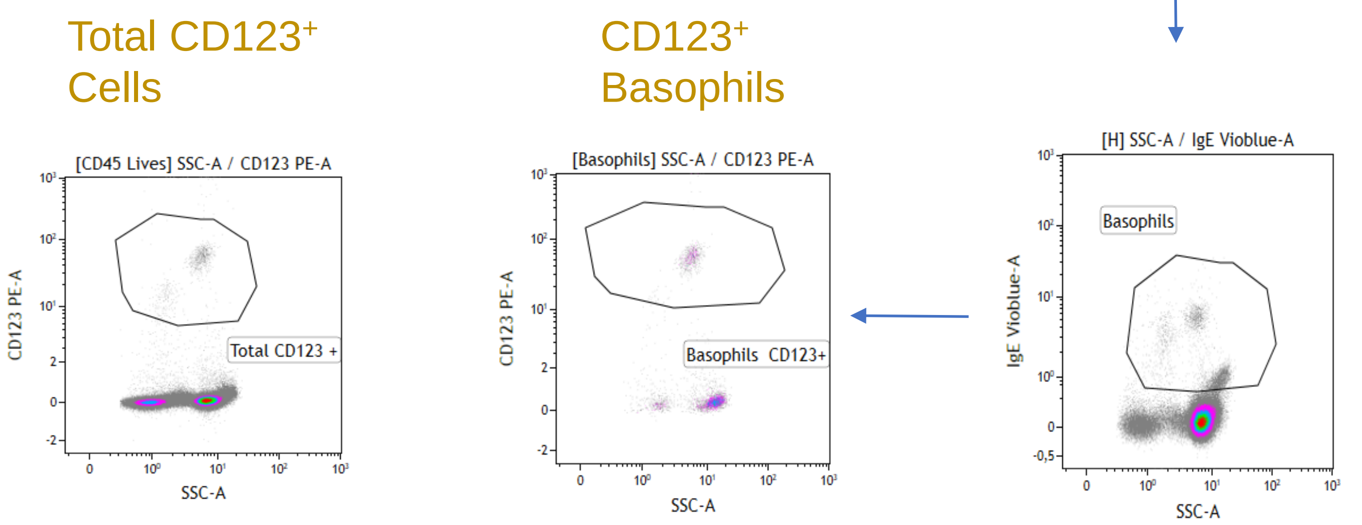

Supplement: Supplementary file 1 — Supplementary Figs. 1–5. [file 41587_2022_1626_MOESM1_ESM.pdf]
